# Supplementary material for: Coevolution-Driven Method for Efficiently Simulating Conformational Changes in Proteins Reveals Molecular Details of Ligand Effects in the β2AR Receptor
Source: J Phys Chem B. 2023 Nov 10;127(46):9891–904. doi: 10.1021/acs.jpcb.3c04897 (PMC10683026; doi:10.1021/acs.jpcb.3c04897)
Supplement: Supplementary file 1 — jp3c04897_si_001.pdf [file jp3c04897_si_001.pdf]

Coevolution-driven Method for Efficiently Simulating Conformational Changes in Proteins Reveals Molecular Details of Ligand Effects in the β2AR Receptor

-

Darko Mitrovic[1,*], Yue Chen[1], Antoni Marciniak[1], Lucie Delemotte[1,*]

1. Department of Applied Physics, Science for Life Laboratory, KTH Royal Institute of Technology, Sweden

* Correspondence to darmi@kth.se and lucied@kth.se

Supporting I~~i~~nformation

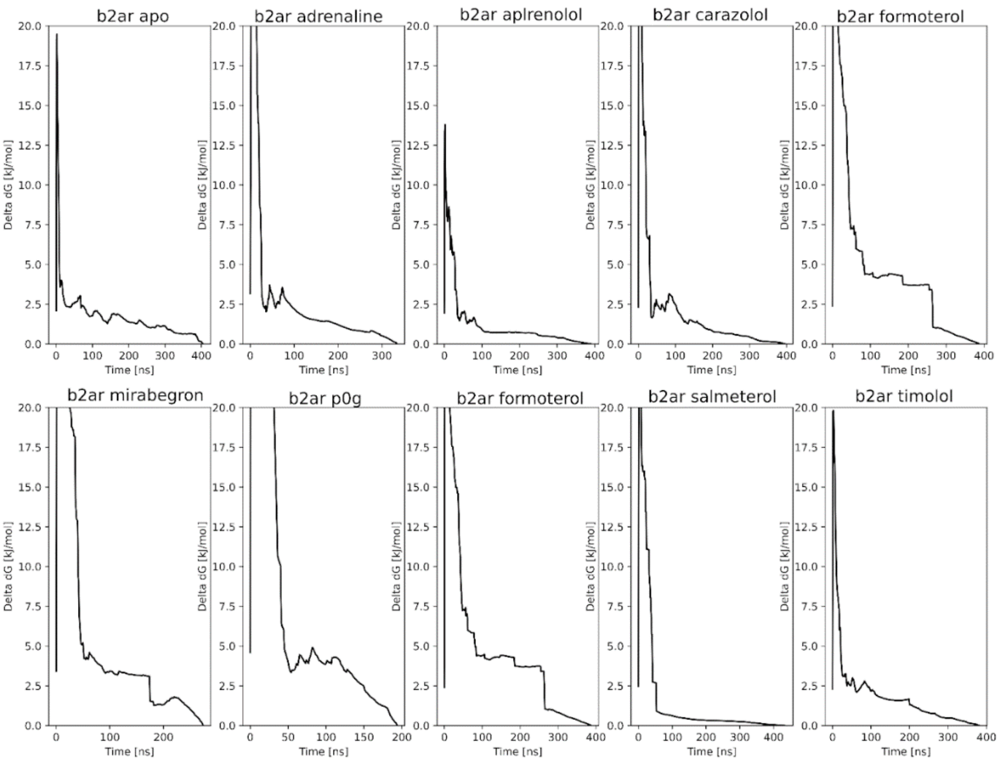

Figure S1. Convergence plots. Each plot shows the ~~mean~~ difference per bin between neighboring simulation frames taken over 1ns intervals over time for all simulation systems. This shows the decrease in free energy estimation in (kJ/mol)/ns over time.

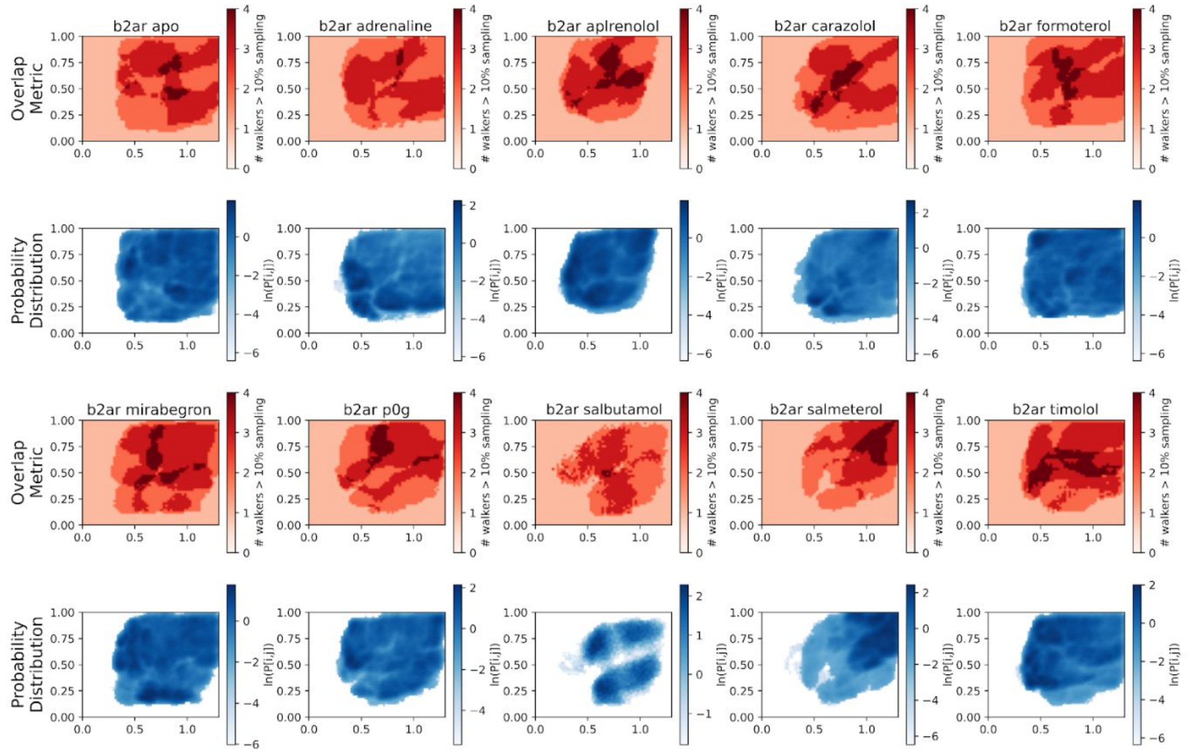

Figure S2. Covering plots. **Top.** The overlap metric, which shows the number of walkers (out of a total of 4 walkers) that cover each point with over 10% of the mean coordinate distribution. **Bottom.** The total probability distribution stemming from all walkers, which shows the unweighted fraction of frames spent over the entire simulation.

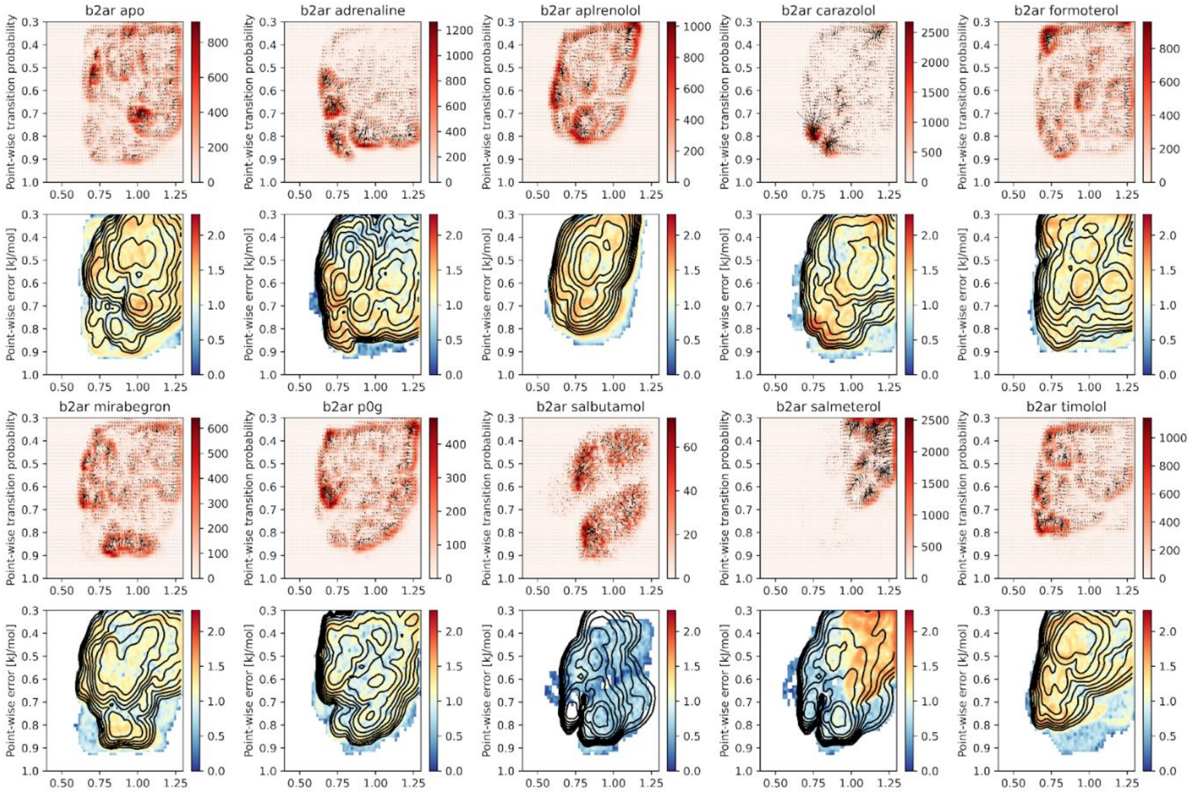

Figure S3. Error plots. **Top.** Vector-shaped transition imbalances colored by the magnitude of the imbalance in raw counts. **Bottom.** The estimated error, calculated as the Boltzmann inversion of the fraction of transition rates between adjacent bins. In this way, a local estimate is shown, representing the magnitude of possible deviation from a perfect bias with a flat probability distribution. The black isocurves represent the free energy surface.

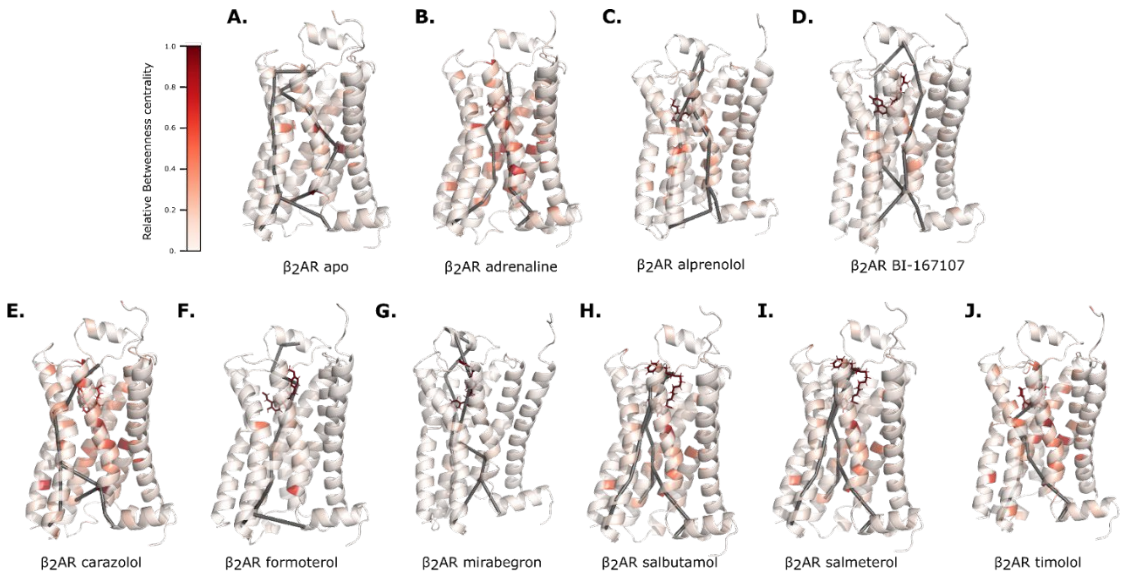

Figure S4. Ligand-wise network analysis. Shortest paths with three main allosteric paths from the ligand binding pocket (F193$^{45.52}$) to the G-protein binding site (E268$^{6.30}$, R131$^{3.50}$, P330$^{8.48}$) calculated with Dijkstra's algorithm and visualized in PyMOL. The networks were constructed with an adjacency matrix with undirected weights calculated as the energetical coupling according to the reweighting scheme.
